# Development of Autoimmune Hair Loss Disease Alopecia Areata Is Associated with Cardiac Dysfunction in C3H/HeJ Mice

**DOI:** 10.1371/journal.pone.0062935

**Published:** 2013-04-26

**Authors:** Eddy Wang, Katy Chong, Mei Yu, Noushin Akhoundsadegh, David J. Granville, Jerry Shapiro, Kevin J. McElwee

**Affiliations:** 1 Department of Dermatology and Skin Science, University of British Columbia, Vancouver, BC, Canada; 2 University of British Columbia, Vancouver, BC, Canada; 3 Department of Pathology and Laboratory Medicine, James Hogg Research Centre, Institute for Heart and Lung Health, University of British Columbia, Vancouver, BC, Canada; 4 Department of Dermatology and Skin Science, Vancouver General Hospital, Vancouver, BC, Canada; Baylor College of Medicine, United States of America

## Abstract

Alopecia areata (AA) is a chronic autoimmune hair loss disease that affects several million men, women and children worldwide. Previous studies have suggested a link between autoimmunity, stress hormones, and increased cardiovascular disease risk. In the current study, histology, immunohistology, quantitative PCR (qPCR) and ELISAs were used to assess heart health in the C3H/HeJ mouse model for AA and heart tissue response to adrenocorticotropic hormone (ACTH) exposure. Mice with AA exhibited both atrial and ventricular hypertrophy, and increased collagen deposition compared to normal-haired littermates. QPCR revealed significant increases in *Il18* (4.6-fold), IL18 receptor-1 (*Il18r1*; 2.8-fold) and IL18 binding protein (*Il18bp*; 5.2-fold) in AA hearts. Time course studies revealed a trend towards decreased *Il18* in acute AA compared to controls while *Il18r1*, *Il18bp* and *Casp1* showed similar trends to those of chronic AA affected mice. Immunohistochemistry showed localization of IL18 in chronic AA mouse atria. ELISA indicated cardiac troponin-I (cTnI) was elevated in the serum and significantly increased in AA heart tissue. Cultures of heart atria revealed differential gene expression between AA and control mice in response to ACTH. ACTH treatment induced significant increase in cTnI release into the culture medium in a dose-dependent manner for both AA and control mice. In conclusion, murine AA is associated with structural, biochemical, and gene expression changes consistent with cardiac hypertrophy in response to ACTH exposure.

## Introduction

The non-scarring hair loss disease alopecia areata (AA) is driven by autoimmune lymphocytes [1], [2]. Over five million people are, or will be, affected by AA in the United States alone, making it one of the most prevalent autoimmune diseases [3]. The development of AA can be associated with other inflammatory diseases such as thyroiditis [4], vitiligo [5], [6] and psoriasis [7]–[9]. While stress as an inducer of AA has long been suspected [7], [10]–[12], studies with rodent models suggest the onset of AA can also exacerbate stress responses. AA affected mice show a decreased ability to cope with physiological stress and a deficit in habituation to repeated psychological stress [13]. Stress hormones such as cortisterone (CORT) and adrenocorticotropic hormone (ACTH) are elevated in AA mice and stress receptors in the brain are altered [13], [14].

Chronic inflammatory conditions can be significant contributing factors to the development of various cardiovascular diseases [15]. The etiology and pathogenesis of chronic inflammatory processes, which usually implicate autoimmune disease, also often involve changes to stress hormone levels [16]. For example, the autoimmune skin disease psoriasis involves chronic upregulation of inflammatory cytokines and dysregulated stress hormones [17]. Development of psoriasis also correlates with an increased risk for atherosclerosis, dilated cardiomyopathy, and myocardial infarction [18], [19]. Potentially, other diseases with chronic inflammation and changes to stress hormone activity, such as AA, could also be associated with heart tissue damage.

Pathologic cardiac hypertrophy is multifactoral; it is common to many cardiovascular diseases such as hypertension and cardiac infarction where the heart increases in muscle mass, but not in contractility, due to dysregulated cardiac remodelling [20], [21]. Several studies have suggested the involvement of stress hormones in cardiac hypertrophy and hypertension; treatment of epilepsy using cortisol and ACTH increases left ventricular mass index [22]–[24].

A possible epidemiological link between AA and heart disease has been suggested, but has not been actively investigated [25], [26]. In this study, we examined the potential structural and molecular changes of hearts in AA affected C3H/HeJ mice. With chronic AA development, cardiac enlargement and other pathological changes were observed. Tissue culture studies suggested exposure to ACTH modulates gene expression and promotes the release of cardiac troponin (cTnI), a marker of heart tissue damage.

## Materials and Methods

### Ethics Statements

All animal studies were approved by the University of British Columbia Animal Care Committee.

### Mice and Tissue Collection

Normal haired, female C3H/HeJ mice (The Jackson Laboratory, Bar Harbor, ME) were induced to express AA by skin grafting as described previously [27]. Age matched sham-grafted littermates as controls. Mice were weighed before euthanasia and their hearts were weighed after blood collection. Mice were euthanized with CO_2_ and subsequent cervical dislocation. Hearts were divided equally such that half was used for RNA extraction while half was used for histology or protein extraction as below.

### RNA Extraction, cDNA Synthesis, and Quantitative Real-Time PCR (qPCR)

RNA extraction from skin and hearts (n = 5) was performed using Qiagen RNeasy Fibrous Tissue Mini Kit (Qiagen, Toronto, ON) with manufacturer’s protocols except double the amount of RLT buffer and Proteinase K were used and QIAshredder (Qiagen) was used to homogenize tissue. First strand cDNA was synthesized from each sample and subjected to reverse transcription using the Superscript first-strand cDNA synthesis kit (Invitrogen, Burlington, ON) according to manufacturer’s protocols using a Mini Cycler (MJ Research, MA).

cDNA templates were mixed with gene specific primers and SYBR Green PCR Master Mix with passive reference dye (Finnzymes, Burlington, ON). Primers (Invitrogen) (Table S1) were designed with Primer3 software [28], *18S* was used as the internal control. The qPCR reactions were completed with an Opticon™ DNA Engine (MJ Research) and always in duplicates. Relative fold change of gene expression in AA mouse tissue compared to controls was calculated as described [29]. Relative quantification was used to determine the fold change in expression of selected target genes in AA mouse tissue compared to sham-grafted (control) derived tissue. A threshold cycle number, ΔC(t), was calculated by normalizing the sample cycle number of the targeted gene with that of the internal control reference gene *18S*. The ΔΔC(t) value was then determined using the formula: ΔΔC(t) = ΔC(t) sample (AA)-ΔC(t)calibrator (normal). The gene expression fold change in AA tissue relative to controls was calculated by 2^-ΔΔC(t)^. Statistical significance (P-value <0.05) was calculated with Student's T test.

### Histology

Masson’s Trichrome Stain (Sigma-Aldrich, Oakville, ON) was used to stain collagen in 6 µm thick Telly-fekete’s acid alcohol fixed heart sections. Cardiac calcinosis has been reported in C3H mice [30]–[32]. Alizarin Red S (Sigma-Aldrich, Oakville, ON) was used to stain calcium deposits. Four hearts each from AA and normal control groups were selected randomly for staining; for each heart, three random sections were analysed and collagen deposition calculated for random blood vessels from each section. The areas encompassed by the collagen, blood vessel lumen, and endothelium were measured in pixels with ImageJ [33] (National Institute of Health, Bethesda, MD). To quantify collagen within whole heart sections, blue pixel (Masson's Trichrome Stain) frequencies were counted with Adobe Photoshop. The hearts were measured randomly to avoid bias. Hematoxylin and Eosin (H&E) staining was performed following standard protocols. Heart sections from AA and control (n = 3 per group) mice were stained and the numbers of nuclei in the atria and ventricles per 100 µm^2^ were quantified and compared with two separate counts for each of the images with atrial and ventricle heart sections; the average of counts from two atrial and two ventricle images were taken for calculation. Cardiomyocyte size in AA and control heart tissue was compared by quantifying the area in pixels encompassed by cardiomyocytes and their corresponding nuclei, and calculating the average nucleus to cardiomyocyte ratio.

### Immunohistochemistry

Immunohistochemistry (IHC) was performed on heart sections from AA (n = 4) and control mice (n = 4) for IL18, IL18R, and IL18BP (all Santa Cruz Biotechnologies, Santa Cruz, CA) using the Vector avidin-biotinylated enzyme complex (ABC) staining system, with Vector red alkaline phosphatase substrate kit (Vector Laboratories, Burlington, ON) and hematoxylin counterstain. Negative controls had no primary antibody.

### Heart Tissue Culture

Atrial tissues from both AA (n = 6) and control mice (n = 6) were divided and treated with four different concentrations of ACTH (0 µM, 0.1 µM, 1 µM, 2 µM) [11], [34]. Tissues were minced into 1 mm blocks and laid in the culture plate. Full length ACTH (Sigma-Aldrich, Oakville, ON) was used (1–39aa) [35]. Serum-free medium, DMEM F12/Glutamax (Invitrogen), was used as described [36] and refreshed every 24-hours with ACTH [37] until 72 hr. QPCR was subsequently performed on RNA extracted from atria (as above) and culture media collected for ELISA analysis (below).

### Total Protein Extraction and Quantification

Hearts from AA affected (n = 4) and control (n = 4) mice were cut into half such that each portion contained one atria and ventricle. Total protein extraction was performed on the atria of one portion of each heart using Total Protein Extraction Kit (Millipore, Billerica, MA) following manufacturer’s protocols. The extracted protein concentration was determined with a BCA protein assay kit (Pierce Biotechnology, Rockford, IL). Standard curves for total protein concentration were calculated and samples equalized.

### Cardiac Troponin I, IL18, IL18R1, IL18BP and CASP1 ELISA

Cardiac troponin I (cTnI), a marker for heart tissue remodelling, is released into the blood stream [38]–[40]. Its expression is increased in those whose heart is undergoing rapid remodelling associated with dysfunction [41]. Mouse cTnI ELISA kits (Life Diagnostics, West Chester, PA) were used to test blood samples of AA (n = 5) and control mice (n = 5). A different cTnI ELISA (Kamiya Biomedical, Seattle, WA) with higher detectable range was used to measure cTnI released in response to ACTH into the supernatant of atria tissue cultures at 72 hours (AA n = 5, control n = 6). 50 ng of total protein from each sample, as determined by BCA protein assay (above), was subjected to cTnI ELISA. A standard curve was generated with the standards provided by the manufacturer and the corresponding equation of the line was used to determine the cTnI concentration in the samples. For mouse IL18 (eBioscience San Diego, CA), IL18R1, IL18BP and CASP1 ELISA (MyBioSource Inc, San Diego, CA), 1 mg of heart tissue homogenate protein was used to perform the assay following the manufacturer’s protocol (n = 3 for each group and time points).

## Results

### AA Mice Displayed Significantly Heavier Heart Weights and Changes in Heart Morphology

**Figure 1:**
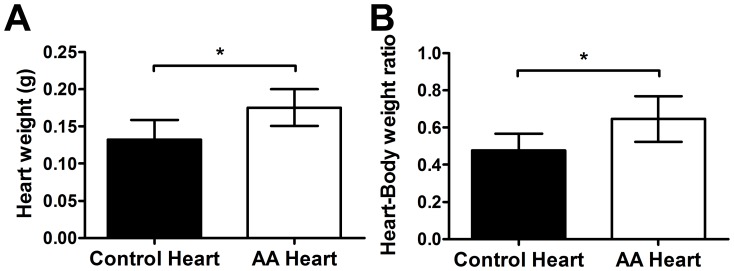
Heart weight and heart to body weight ratio in AA and sham-grafted mice. The wet heart weight (A) and heart to body weight ratio (B) of AA (n = 10) and healthy sham-grafted mice (n = 11) revealed significantly heavier hearts in AA mice. Statistical significance was determined with Student’s t test where *denotes p≤0.05.

**Figure 2:**
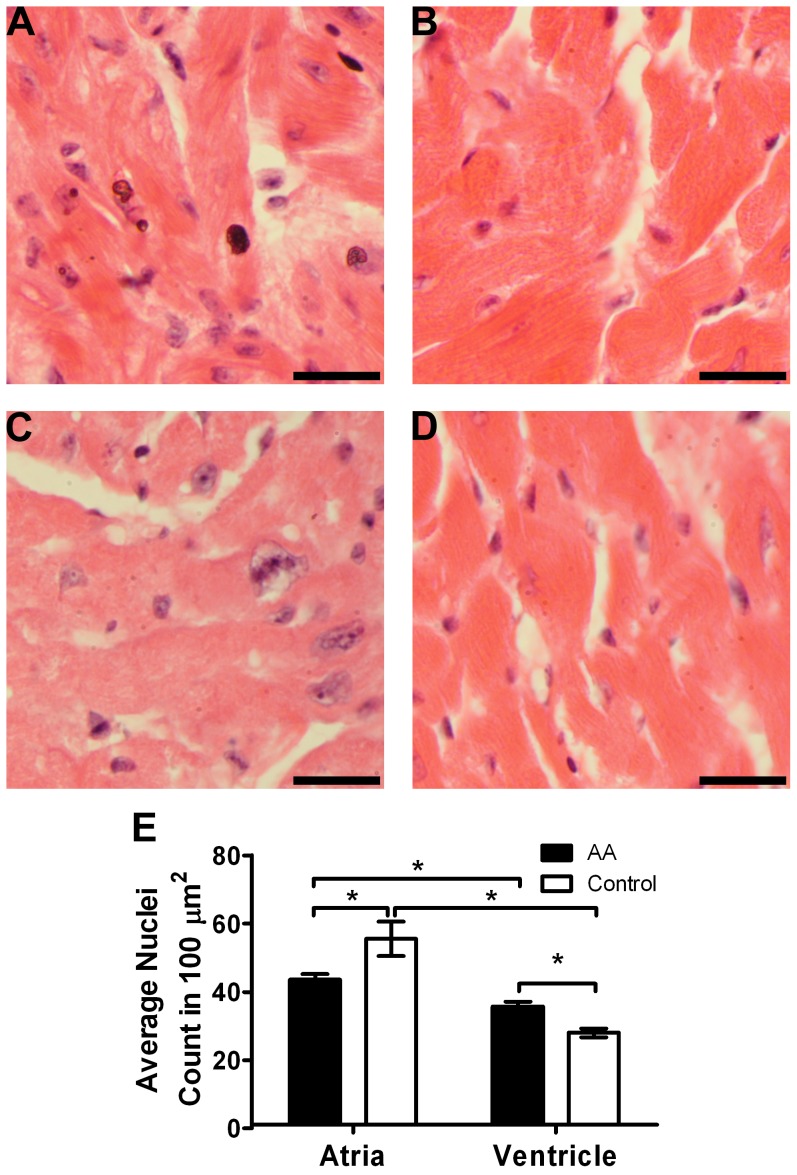
AA mice had significantly fewer nuclei in atria tissue compared to sham-grafted mice. Sham-grafted control mouse atria (A) and ventricles (B) and AA mouse atria (C) and ventricles (D) were H&E stained. The number of nuclei in 100 µm^2^ was quantified (E). AA mice had significantly fewer nuclei in their atria but significantly more nuclei in their ventricles compared to the controls. Statistical significance was determined with Student’s *t*-test where *denotes p<0.05. Bar = 20 µm.

There was a significant difference between the heart weights of AA mice and normal mice (Figure 1A). The heart to body weight ratio of AA mice was also significantly greater than the control mice (Figure 1B). The changes in heart morphology were identified with H&E staining (Figure 2A–D). There were significantly lower frequencies of nuclei in the atria of AA mouse hearts compared to the controls despite the increase in heart size (Figure 2E). However, the ventricles of AA mice had significantly higher frequencies of nuclei compared to the controls (Figure 2E). The average area encompassed by the atrial cardiomyocytes and their corresponding nuclei and also nucleus to whole cell area ratio (0.147 in AA, 0.095 in control) was greater in AA mice compared to control mice; with statistically significant differences achieved for the average area of the nucleus and nucleus to whole cell area ratios (both p<0.0005).

### Increased Collagen Deposition in AA Hearts

**Figure 3:**
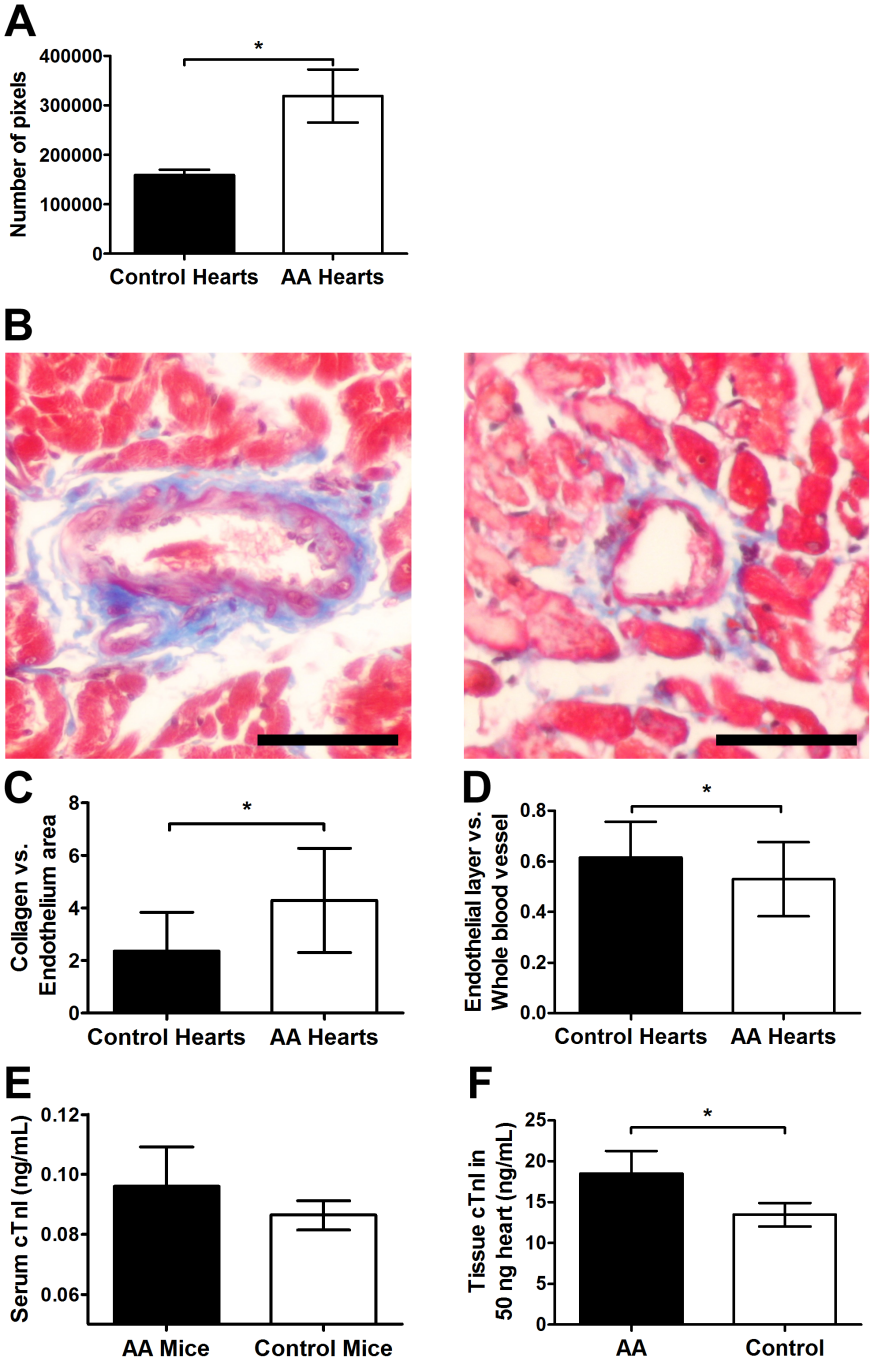
Evaluation of heart collagen deposition and cardiac troponin-I (cTnI) in AA and sham-grafted mice. Areas encompassed by collagen, lumen, blood vessels, and blood vessels plus surrounding collagens, were measured with ImageJ (A, C, D). There was a higher amount of collagen infiltration into the blood vessels within the hearts of AA mice (B, AA left; Control right). AA mice had significantly larger regions of collagen deposition around blood vessels but had significantly thinner blood vessel wall thickness compared to normal controls. Three random readings performed from each of 3 different non-consecutive slides per mouse for AA (n = 4) and control (n = 4) mice. ELISA revealed a higher amount of serum cTnI associated with AA (n = 5 per group) compared to control mice, though not statistically significant (E). The concentration of cTnI in the heart tissue of AA mice was significantly higher than the control mice (F). Statistical significance was determined with Student’s *t*-test where *denotes p<0.05. Bar = 50 µm.

The total amount of collagen in AA mouse hearts was significantly higher than control hearts (Figure 3A). Collagen was largely restricted to the periphery of blood vessels (Figure 3B). The average ratio of areas encompassed by collagen versus areas encompassed by blood vessel walls (endothelial layer) was significantly higher in AA mice (Figure 3C); indicating increased collagen accumulation. The average width of heart blood vessel walls in AA mice was also found to be significantly lower than in healthy controls (Figure 3D). Taken together, AA mouse hearts presented with increased peri-vascular collagen. Calcinosis was minimal and not different between AA mice and controls (not shown).

### AA Affected Mice have Higher Concentrations of cTnI in Heart Tissue and Plasma

As cTnI is a cardiac regulatory protein of actin and myosin interaction [42], the elevation of plasma cTnI levels is an indication of myocardial tissue insult [42], [43]. ELISA revealed a trend for higher cTnI levels in AA affected mice compared to controls (Figure 3E) potentially consistent with an overall deterioration of heart health. AA mice also had significantly higher concentrations of cTnI in heart protein homogenates compared to healthy controls (Figure 3F) consistent with higher serum cTnI. This indicates that, despite the low gene expression (Figure S1B), there was still a relatively high level of cTnI protein.

### Pro-inflammatory Cytokine Gene Expression is Significantly Higher in AA Mouse Skin and Heart Tissues

**Figure 4:**
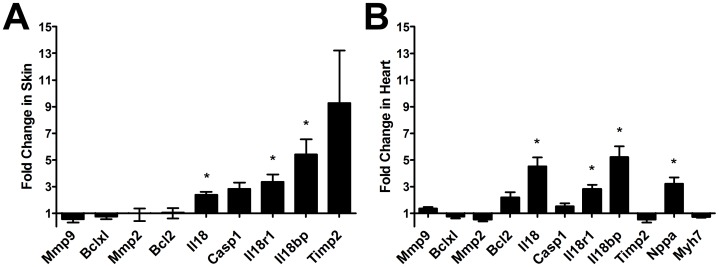
qPCR analysis of selected genes in AA and sham-grafted mice in chronic stage. In an initial screen for various heart disorder related gene markers, there was a significant increase of *Il18, Il18r1 and Il18bp* gene in both skin (A) and hearts (B) of AA mice (n = 4) compared to sham-grafted control mice (n = 5). The expression of *Nppa* was also significantly increased in the hearts of AA mice. QPCR relative fold change in gene expression analyses were calculated using 2^−ΔΔCt^; average fold change is presented. Error bars represent the range factor difference (2^−ΔΔCt±ΔCtSD^). Statistical significance determined with Student’s *t*-test; *denotes p<0.05.

We conducted a preliminary gene expression screen for diverse genes implicated in heart disease tissue damage which first identified pro-inflammatory cytokine Interleukin 18 (*Il18*) (Figure S1B). Subsequently, qPCR analysis in AA mice revealed *Il18*, *Il18* receptor-1 (*Il18r1*) and *Il18* binding protein (*Il18bp*) to be significantly increased in their heart tissues; 2.8, 4.5 and 5.2 fold respectively compared to control mice, 18 months after skin grafting (Figure 4A,B). Markers of heart hypertrophy were also examined. While changes in β-myosin heavy chain (*Myh7*) were insignificant, the mRNA expression for atrial natriuretic factor (*Nppa*) was significantly increased 3.2 fold in AA mouse hearts compared to controls (Figure 4B).

### Changes in Gene and Protein Expression during the Onset of AA

**Figure 5:**
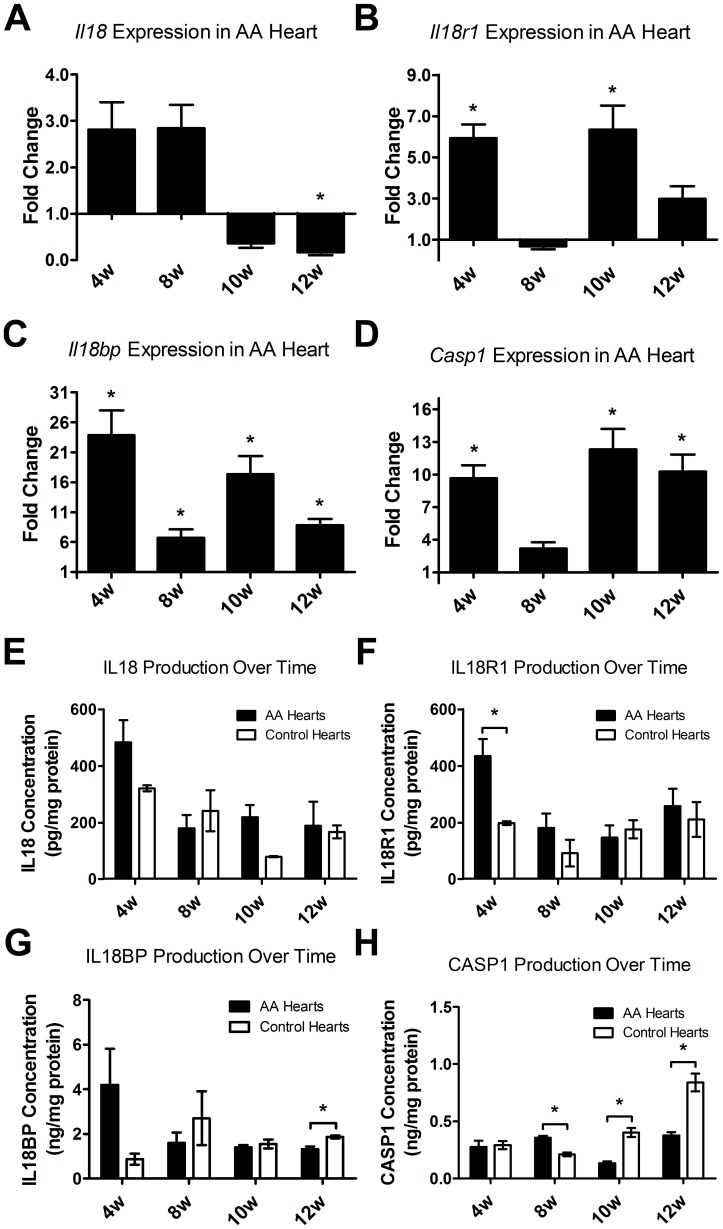
qPCR and ELISA analysis of *Il18* and related genes and protein expression in AA and sham-grafted mice during the onset of AA. Comparing AA mice to sham grafted mice (n = 3 per time point), *Il18* gene expression fold change in mouse hearts showed an increase in the expression pattern before the onset of AA, but decreased expression around the time of AA onset. *Il18r1*, *Il18bp* and *Casp1* (A–D) showed mostly significant elevation both before and after onset of AA. At the protein level, the expression of IL18, IL18R1 and IL18BP displayed similar trends as with qPCR (E–G). However, *Casp1* expression was significantly higher in sham grafted mice at 10 and 12 weeks (H). QPCR analyses for gene expression levels were calculated as fold change by using the 2^−ΔΔCt^, average fold change presented. Error bars represent the range factor difference (2^−ΔΔCt±ΔCtSD^). Statistical significance determined with Student’s *t*-test; *denotes p<0.05.

Mice usually develop AA around 10 weeks after skin grafting [27]. The gene expression profile dynamics of *Il18*, *Il18r1*, *Il18bp*, and Caspase-1 (*Casp1*) in hearts were investigated in response to AA skin grafts (Figure 5A–D). Compared to sham-grafted control mice, qPCR analysis revealed increased *Il18* expression shortly after skin grafting prior to overt hair loss, but expression decreased when the mice first started to lose hair around 10 weeks after grafting. The expression of *Il18bp* was increased in AA mouse hearts compared to controls throughout the first 12 weeks after skin grafting; before and after hair loss onset. Both *Il18r1* and *Casp1* maintained significantly increased expression in AA mice within the first 12 weeks except just before the onset of AA (eight weeks). Overall, *Il18bp*, *Il18r1* and *Casp1* all showed similar increased expression patterns as observed in chronic AA affected mice. The protein expression of IL18, IL18R1 and IL18BP as evaluated by ELISA showed similar trends to gene expression in the hearts (Figure 5E–H). IL18, IL18R1 and IL18BP showed decreasing trends as AA began to develop around eight weeks. At 12 weeks, IL18BP in AA mouse hearts expressed significantly lower IL18BP compared to the controls. CASP1 expression in AA mouse hearts was significantly decreased at 10 and 12 weeks.

### The Expression of IL18 is Localized in the Atria of AA Mouse Hearts

**Figure 6:**
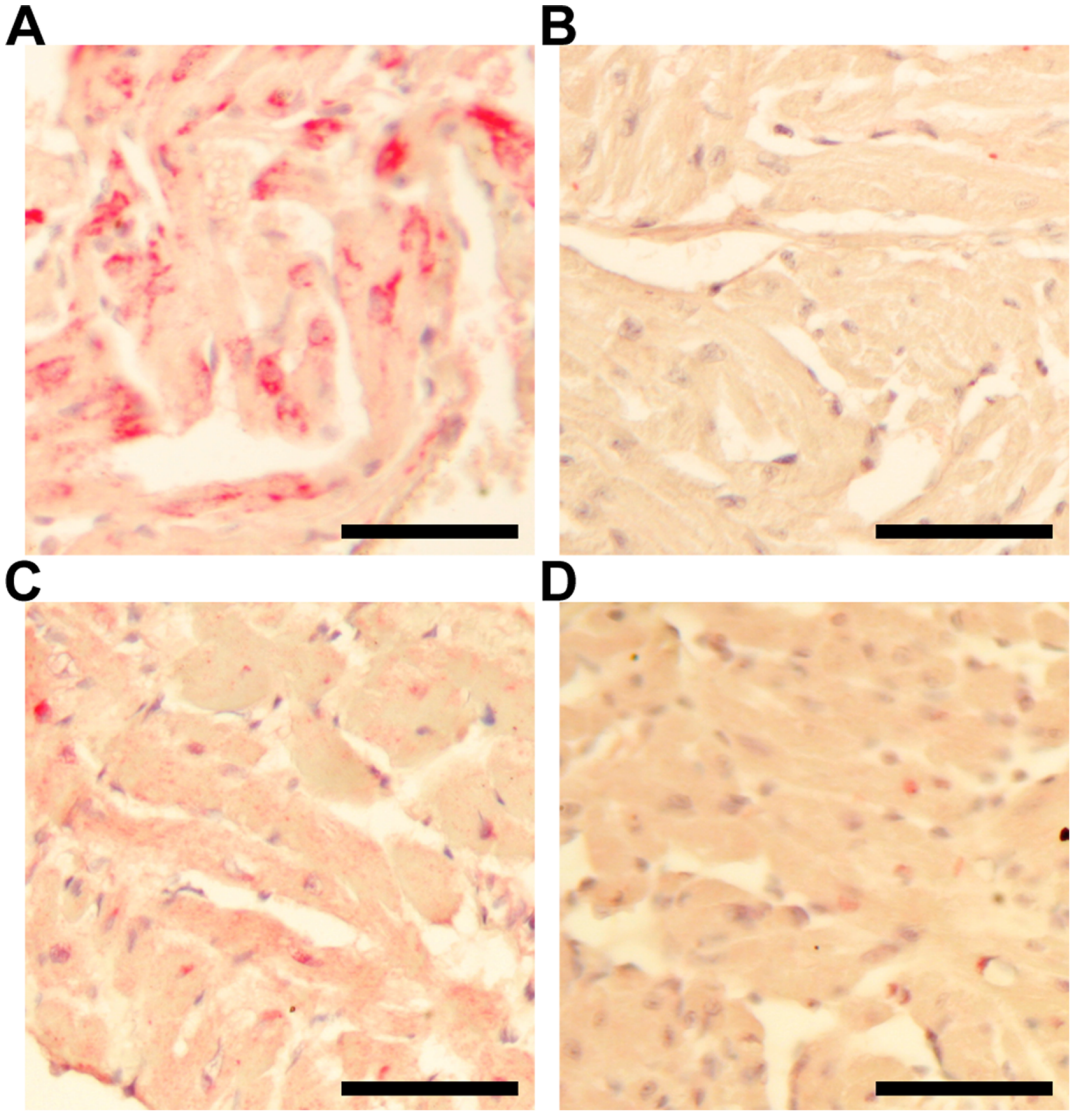
Immunohistochemistry of IL18 on AA and sham-grafted mice. IHC performed on AA and sham-grafted mice heart sections revealed localized expression of IL18 (red) in the atria of AA mice (A). In healthy sham-grafted mice, there was no specific labeling in the atria of the heart (B). In both groups of mice, no specific labeling for IL18 was found in the ventricles. AA mouse hearts (C) had slightly more specific expression of IL18R1 near the apical part of the heart compared to healthy controls (D). Three random paraffin-embedded slides from each mouse, each containing 3 to 4 sections, were analyzed in parallel. Slides were counterstained with hematoxylin (blue). Bar = 50 µm.

By immunohistochemistry (IHC) all AA mice displayed an atrial-specific localization of IL18 (Figure 6A) while no specific labelling was found in the ventricles, or in the hearts of control mice (Figure 6B). The expression pattern for IL18R1 was similar when comparing AA to sham-grafted controls (Figure 6C, D). The IL18 antagonist, IL18BP, displayed weak and unspecific expression in both AA and controls, indicating a possible low protein expression despite the increased gene expression (not shown).

### Differential Response of Gene Expression between AA and Control Mouse Atria with ACTH Treatment in Culture

**Figure 7:**
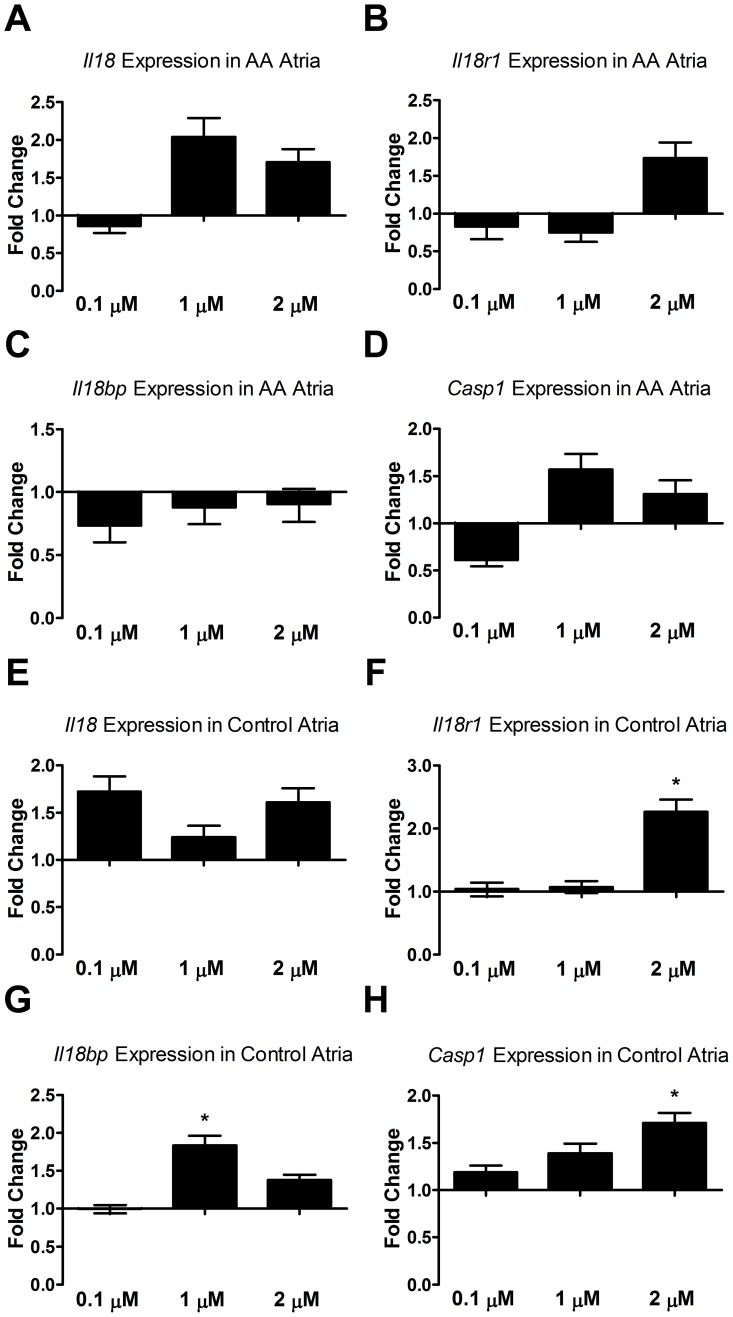
qPCR analysis of atria treated with ACTH for 72 hours. For atria derived from AA mice, *Il18* expression was highest at 1 µM of ACTH (A); the expression of *Casp1* showed similar trend (D). *Il18r1* expression was dependent on ACTH concentration (B) while *Il18bp* was down-regulated (C). For atria derived from sham-grafted mice, *Il18* expression was lowest at 1 µM unlike AA (E). However, *Il18bp* was significantly increased at 1 µM (G). ACTH had a significant effect on the control atria with concentration dependent increase of *Il18r1* (F) and *Casp1* (H). Gene expression levels were calculated as fold change compared to no-treatment control (0 µM ACTH). Error bars represent the range factor difference (2^−ΔΔCt±ΔCtSD^). Statistical significance determined with Student’s *t*-test; *denotes p<0.05.

The stress hormone ACTH promotes IL18 secretion via the modulation of caspase-1 in the adrenal gland during stress [44]–[46], but its effect on IL18 secretion in the heart is unknown. With ACTH exposure there was an increase in *Il18*, *Il18r1*, and *Casp1* expression compared to the no-treatment control in the atria of AA mice, but data were not statistically significant (Figure 7A–D). However, ACTH statistically significantly increased *Il18bp*, *Il18r1*, and *Casp1* in control mouse atria (Figure 7E–H). Notably, the expression pattern for *Il18r1* and *Ice* in control mouse atria both showed a concentration dependent increasing trend, reaching statistical significance at 2 µM ACTH.

### Increased Expression of Major Collagen Genes in the Atria with ACTH Treatment

**Figure 8:**
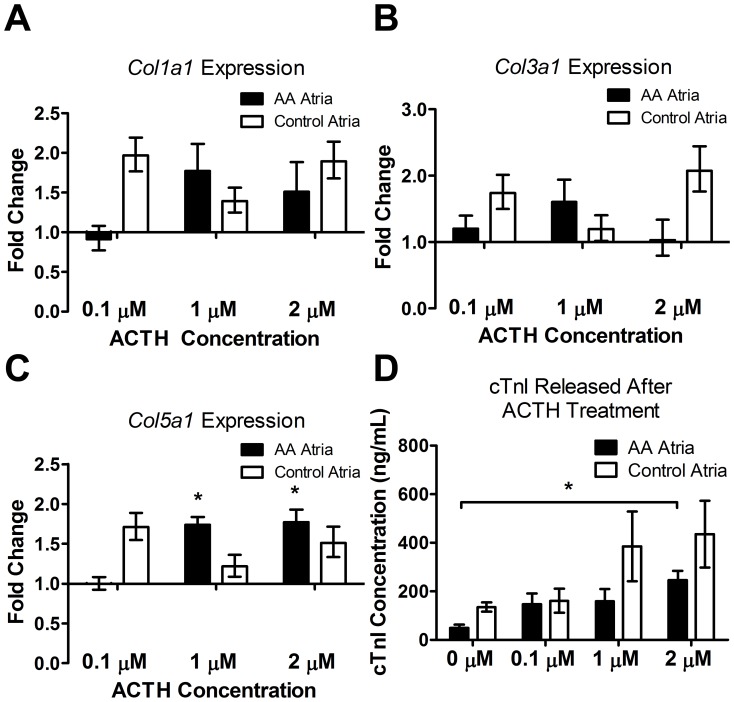
Gene expression of collagens and the release of cardiac troponin from AA and sham-grafted mouse atria in response to ACTH treatment after 72 hours. Both AA and control mice displayed ACTH concentration dependent increase of *Col1a1* (A), *Col3a1* (B), *Col5a1* (C) and the release of cTnI from atria (D). However, significant increase was only observed in AA mouse atria for *Col5a1* (at 1 µM and 2 µM ACTH) and release of cTnI (at 2 µM ACTH) compared to no-treatment control. QPCR analyses for gene expression levels were calculated as fold change by using the 2^−ΔΔCt^, average fold change presented. Error bars represent the range factor difference (2^−ΔΔCt±ΔCtSD^). For cTnI ELISA, a standard curve was generated with the standards provided by the manufacturer and the corresponding equation of the line was used to determine the cTnI concentration in the sample. Statistical significance determined with Student’s *t*-test; *denotes p<0.05.

The gene expression of *Col1a1* (Collagen Ia1), *Col3a1* (Collagen IIIa1) and *Col5a1* (Collagen Va1) in the atria of AA and normal mice (both n = 3) was evaluated by qPCR. All collagens showed an ACTH concentration dependent increase; at both 1 µM and 2 µM of ACTH, the expression of *Col5a1* was significantly increased in AA mice (Figure 8A–C).

### Cardiac Troponin I Increased in Both AA and Control Mouse Atria with Increasing ACTH Concentration

ELISA quantification of cTnI revealed both AA and control mouse atrial tissues released more cTnI into the culture medium after ACTH treatment and the increase was dose dependent (Figure 8D). The release of cTnI from AA mouse atria was significantly increased at 2 µM of ACTH compared to no treatment controls.

## Discussion

A relationship between androgenetic alopecia, cardiovascular disease and hypertension has been demonstrated by various groups, though the exact biological mechanism remains elusive [47]–[50]. Potential relationships between other forms of hair loss and heart tissue damage have not been actively investigated beyond brief epidemiological reports. In this study, hearts were found to be significantly enlarged in AA mice compared to healthy, sham-grafted littermates (Figure 1). The variable extent of hair loss and its fluctuation over time may affect (and potentially reflect) the degree of heart damage, consistent with the high variability of results between each mouse. C3H/HeJ mice are relatively resistant to atherosclerosis [51]; potentially this may alter their pathological presentation.

Further investigations into changes in heart morphology (Figure 2A–D) revealed a significant difference between the density of cardiomyocyte nuclei in the atria and ventricles of AA mice compared to the controls (Figure 2E). AA mouse atria had significantly fewer nuclei per unit area compared to the controls. Cardiomyocytes in AA hearts presented with statistically significantly larger nuclei and a greater nucleus to whole cell area ratio in AA mice compared to controls, consistent with atrial hypertrophy [52]. In contrast, the frequency of nuclei in the ventricles of AA mouse hearts was significantly higher than the controls.

AA hearts had an overall statistically significantly higher amount collagen compared to normal hearts (Figure 3A,B). The blood vessel walls of AA mouse hearts were also significantly thinner than those of sham-grafted mice (Figure 3D). It has been shown that elevated peri-vascular collagen is a pathological event in many forms of cardiovascular disease [53], [54] and can increase the stiffness and decrease the contractility of the heart [55]. Potentially, the thinning of the blood vessel walls may be a result of collagen reorganisation by cardiac fibroblasts [56]. Such blood vessel wall thinning may also be a result of endothelial cell size decrease, a phenomenon observed in hypertension [57]. The accumulation of extracellular matrix (ECM) can modulate cellular function and size; type I, III, IV collagen and fibronectin can decrease aortic endothelial cell migration, proliferation and size [58]. Increased collagen around and within blood vessel walls (Figure 3C) can lead to hypertrophy and ultimately to heart failure [59].

*Il18*, *Il18r1*, and *Il18bp* were significantly increased AA mouse hearts (Figure 4B). Increased *Il18r1* may have a synergistic effect with *Il18* by increasing the sensitivity to the ligand [60]. The expression of *Casp1* was also elevated; increased caspase-1 may increase the amount of activated form of IL18. IL18BP protein is an antagonist of IL18; its gene expression alongside *Il18* and *Il18r1* may be a sign of an activated negative-feedback system to counter adverse IL18 activity [61]. A marker for cardiac hypertrophy, *Nppa*, was also found to be significantly increased in the AA mouse hearts (Figure 4B). Nppa is a vasodilator released by the atrial tissues in response to stretch and remodelling; its expression can be induced by IL18 [62]. Increased IL18 product intensity and localization was found in AA mouse atria (Figure 6A). This suggests that AA may be associated with heart inflammation and/or defects in the regulation of inflammation. Injection of IL-18 into mice induces myocardial hypertrophy and heart remodeling [63]. Therefore, the elevation of *Il18* expression in the heart, in conjunction with the abnormal hypertrophy in AA affected mice, may be a marker of inflammatory heart disease similar to observations with dilated cardiomyopathy [64], [65].

IL18 can induce interferon γ (IFNγ) production from lymphocytes and natural killer cells [65]–[70]. It has been shown to be involved in cardiovascular diseases such as ischemia-reperfusion injury, and atrial fibrillation [64], [71]–[73], where it induces cell mediated inflammation and myocardial fibrosis [74]–[77]. However, we did not observe inflammatory cell infiltration in AA hearts. Alternatively, dysfunctional caspase-1 activity can lead to increased secretion of IL18 and IL1β in heart-specific “auto-inflammatory disease” in the absence of cell infiltration [78], [79]. It is also possible that AA activated lymphocytes release IL18 into circulation as observed in other autoimmune diseases [80]. Increased IL18 has been found in AA patient plasma [81]. Increased IL18 activity in the heart could lead to cardiac hypertrophy and increased cell apoptosis without direct lymphocyte infiltration [65]. However, the exact link between IL18 and the changes in the hearts of AA mice remains to be determined.

The serum level of cTnI, a myocardial regulatory protein that is elevated after cardiac injury [82], [83], was higher in AA affected mice compared to sham-grafted controls (Figure 3E). Though not statistically significant, the trend observed in the serum is consistent with heart tissue remodelling. The release of cTnI can precede the actual onset of more severe forms of heart disease and can serve as a hypertension marker [83]. The significantly higher levels of cTnI in AA heart tissue compared to control mice (Figure 3F) is consistent with tissue remodelling and heart hypertrophy and thus increased demand for cTnI. The gene expression of cTnI may be limited by the expression of other molecules such as, IGFBP, as one of the ways to inhibit heart hypertrophy [84].

Adrenocorticotrpic hormone (ACTH) is a known inducer of IL18 [44], [46]. Evidence suggests that there is abnormal regulation of stress hormones and receptors in AA affected mice [13], [14], [85]. AA mice display higher levels of ACTH and corticosterone, possibly due to the inflammatory cytokines released by the lymphocytes involved in AA development [13]. With ACTH exposure, we found that *Il18*, *Il18r1*, and *Casp1* gene expression increased in mouse atria and statistical significance was achieved with normal mouse atria (Figure 7E–H). Possibly, as AA mouse atria were likely previously exposed to increased ACTH activity *in vivo*, there was limited opportunity for even greater modulation of gene expression by ACTH in our *in vitro* assay. Sham-grafted mice may be somewhat better at coping with the effect of increased ACTH by increasing the expression of *Il18bp*. However, the net result suggests ACTH can promote IL18 activity.

With ACTH exposure, the gene expressions for various collagens increased in both AA and control mouse atria. ACTH has been shown to increase bone mass by increasing the production of type I collagen in osteoblast cell lines [86]–[89]. In AA mice, their elevated ACTH levels could potentially increase collagen production in the heart resulting in adverse effects. *Col1a1*, *Col3a1* and *Col5a1* gene expression were all increased in AA mouse atria while less pronounced in control mice. Type V collagen is a regulator of Type I collagen assembly [90] and the significant increase of the Type V collagen gene (*Col5a1*) in AA mice may be consistent with excess accumulation of type I collagen [91].

Stress hormones are associated with damage to the cardiovascular system, though the exact mechanism is unclear [92], [93]. Excess Type V collagen production in response to ACTH is one possible hypothesis. Also, ACTH is reported to suppress the inactivation of cortisol, which may have inflammatory effects on the vasculature and result in hypertension [94]. Injury was confirmed by measuring the amount of cTnI released into culture medium after 72 hours. Culture medium cTnI levels increased after ACTH exposure for both AA and sham-grafted mouse atria indicating heart tissue changes in response to ACTH. AA atria released significantly more cTnI (Figure 8D) suggesting AA mouse atria may be more susceptible to ACTH action compared to control mice.

The results presented emphasize that AA is not just restricted to the hair follicles. The sequelae of AA development may have impact on other tissues and organs beyond the skin. We have provided evidence that AA development in mice is associated with abnormal heart hypertrophy, associated with elevation of *Il18*, *Col5a1* and cardiac remodelling marker, cTnI. Stress hormones, such as ACTH, can accentuate the production of *Il18* and may lead to damage in the heart and the release of cTnI. The results presented in this study suggest that AA onset can be a predisposing factor to abnormal heart remodelling and closer follow-up for patients with AA should be considered.

## Supporting Information

Figure S1 Preliminary qPCR gene screening of chronic AA mice compared to the healthy controls. In both the skin (a) and heart (b), there was a significant increase of *Il18* and significant decrease of *Cti* in the AA mice (n = 6) compared to the healthy sham-grafted controls (n = 6). There was an over 1,000 fold increase in granzyme B (*Gzmb*) activity in the skin of AA mice but such increase was not observed in the heart. Statistical significance was determined with Student’s *t* test where *denotes p<0.05.
(DOC)

Table S1 Primer sequences used for qPCR analysis.
(DOC)
