# Supplementary figures and images for: Development of Autoimmune Hair Loss Disease Alopecia Areata Is Associated with Cardiac Dysfunction in C3H/HeJ Mice
Source: PLoS One. 2013 Apr 26;8(4):e62935. doi: 10.1371/journal.pone.0062935 (PMC3637254; doi:10.1371/journal.pone.0062935)

**
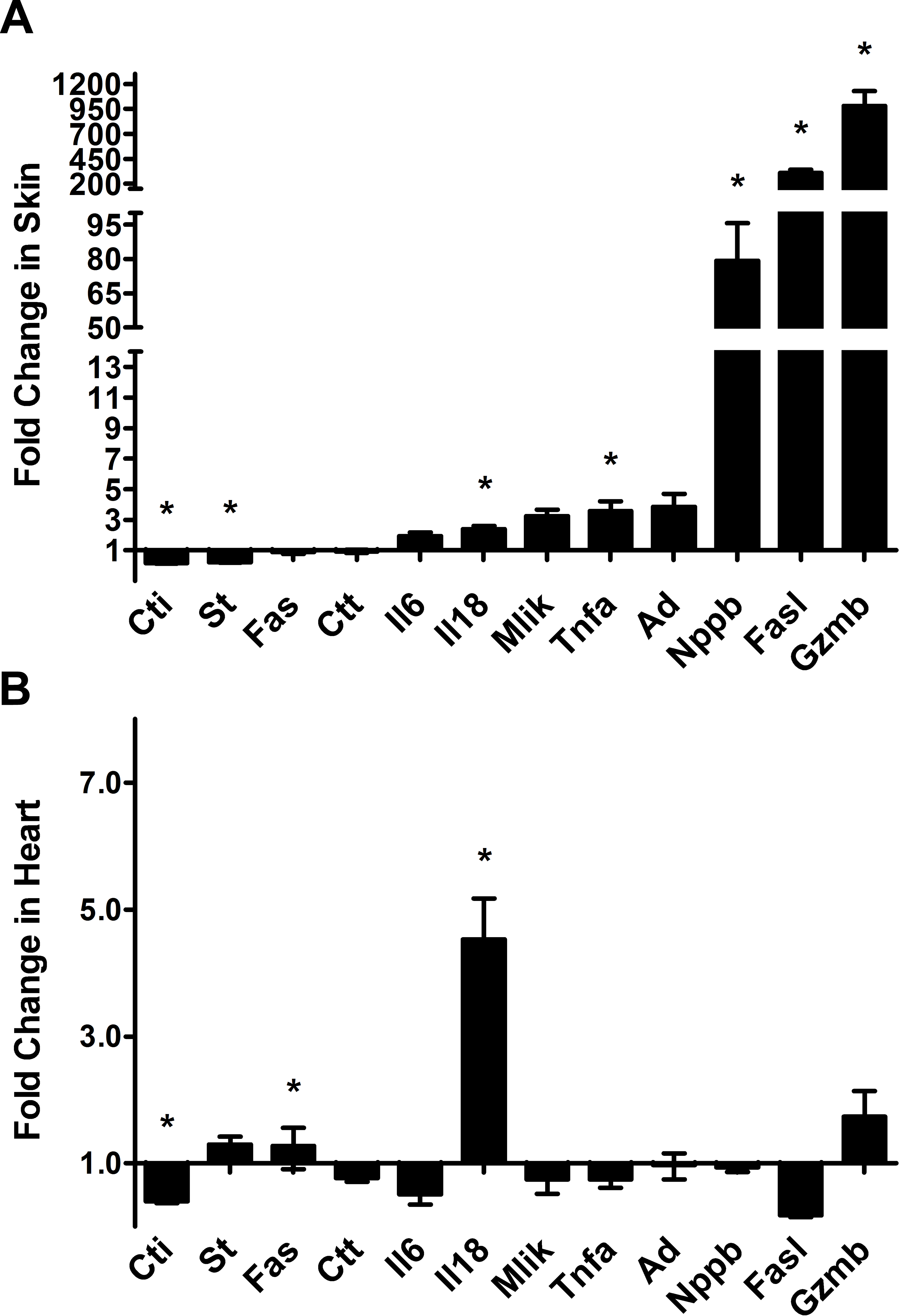
**

Supplement: Figure S1 — Preliminary qPCR gene screening of chronic AA mice compared to the healthy controls. In both the skin (a) and heart (b), there was a significant increase of Il18 and significant decrease of Cti in the AA mice (n = 6) compared to the healthy sham-grafted controls (n = 6). There was an over 1,000 fold increase in granzyme B (Gzmb) activity in the skin of AA mice but such increase was not observed in the heart. Statistical significance was determined with Student’s t test where *denotes p<0.05. (DOC) [file pone.0062935.s001.doc]
