# Supplementary material for: Development of Autoimmune Hair Loss Disease Alopecia Areata Is Associated with Cardiac Dysfunction in C3H/HeJ Mice
Source: PLoS One. 2013 Apr 26;8(4):e62935. doi: 10.1371/journal.pone.0062935 (PMC3637254; doi:10.1371/journal.pone.0062935)
Supplement: Table S1 — Primer sequences used for qPCR analysis. (DOC) [file pone.0062935.s002.doc]

| **Gene** | **Symbol** | **Forward** | **Reverse** |
| --- | --- | --- | --- |
| Cardiac Troponin I | *Cti* | TCTGCCAACTACCGAGCCTAT | CTCTTCTGCCTCTCGTTCCAT |
| Cardiac Troponin T | *Ctt* | CAGAGGAGGCCAACGTAGAAG | TCGATCAGAGTCTGTAGCTCATT |
| Fas | *Fas* | TGCAAGTGCAAACCAGACTTC | GTCAACAACCATAGGCGATTTCT |
| Fas Ligand | *Fasl* | ACCCCCACTCAAGGTCCAT | CGAAGTACAACCCAGTTTCGT |
| St2 (IL1 Receptor-like 1) | *St2* | ATGGGAGAGACCTGTTACCTG | CCTGCTCGTAGGCAAATTCCT |
| Myosin light polypeptide kinase | *Mlik* | TGGGGGGACGTGAAACTGTTTG | GGGGCAGAATGAAAGCTGG |
| Interleukin 6 | *Il6* | TCCAGTTGCCTTCTTGGGAC | GTGTAATTAAGCCTCCGACTTG |
| TNFα | *Tnfa* | TTCTGTCTACTGAACTTCGGGGTGATCGGTCC | GTATGAGATAGCAAATCGGCTGACGGTGTGGG |
| Adrenomedullin | *Ad* | GGAATAAGTGGGCGCTAAGTC | CAAGAGTCTGGGTAGGAACTGT |
| Natriuretic peptide type B | *Nppb* | CCCAAAAGAGTCCTTCGGTC | CGGTCTATCTTGTGCCCAAAG |
| Granzyme B | *Gzmb* | TGGACCCTACATGGCCTTAC | TGGGGAATGCATTTTACCAT |
| Interleukin 18 | *Il18* | GTGAACCCCAGACCAGACTG | CCTGGAACACGTTTCTGAAAGA |
| Matrix metallopeptidase 2 | *Mmp2* | TTTGCTCGGGCCTTAAAAGTAT | CCATCAAACGGGTATCCATCTC |
| Matrix metallopeptidase 9 | *Mmp9* | TGCCCATTTCGACGACGAC | GTGCAGGCCGAATAGGAGC |
| IL18 Receptor-1 | *Il18r1* | TAATCATCGTTCTCAGCCAGAGT | GGACTGTCAGCCCTCCATTTT |
| IL18 Binding Protein | *Il18bp* | CCTACTTCAGCATCCTCTACTGG | AGGGTTTCTTGAGAAGGGGAC |
| B cell lymphoma 2 | *Bcl2* | GCTACCGTCGTGACTTCGC | CAACCAGACATGCACCTACCC |
| BCL2-like 1 | *Bclxl* | GGGATGGAGTAAACTGGGGTC | TGTTCCCGTAGAGATCCACAAA |
| Tissue inhibitor of metalloproteinase 2 | *Timp2* | TCAGAGCCAAAGCAGTGAGC | GCCGTGTAGATAAACTCGATGTC |
| Caspase 1 | *Casp1* | ACAAGGCACGGGACCTATG | TCCCAGTCAGTCCTGGAAATG |
| Collagen Ia1 | *Col1a1* | AGCTTTGTGGACCTCCGGCT | ACACAGCCGTGCCATTGTGG |
| Collagen IIIa1 | *Col3a1* | TGCCCACAGCCTTCTACACCT | CCAGCTGGGCCTTTGATACCT |
| Collagen Va1 | *Col5a1* | TGAATTCAAGCGTGGGAAACT | CCGCAGGAAGGTCATTTGTAC |
| Atrial Natriuretic Factor | *Nppa* | GTACAGTGCGGTGTCCAACA | TCTCCTCCAGGTGGTCTAGCA |
| β Myosin Heavy Chain | *Myh7* | GCATTCTCCTGCTGTTTCCTT | TGGATTCTCAAACGTGTCTAGTGA |
